# Supplementary figures and images for: A Customizable Low-Cost System for Massively Parallel Zebrafish Behavioral Phenotyping
Source: Front Behav Neurosci. 2021 Jan 18;14:606900. doi: 10.3389/fnbeh.2020.606900 (PMC7847893; doi:10.3389/fnbeh.2020.606900)

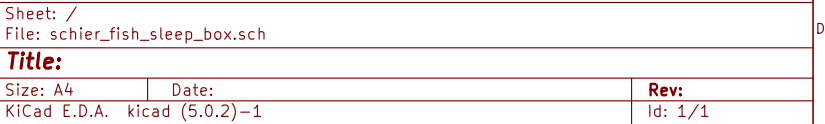

Supplement: Supplementary file 3 [file Data_Sheet_2.ZIP › FabricationFiles/PCB/schematic.pdf]
