# Supplementary material for: A Customizable Low-Cost System for Massively Parallel Zebrafish Behavioral Phenotyping
Source: Front Behav Neurosci. 2021 Jan 18;14:606900. doi: 10.3389/fnbeh.2020.606900 (PMC7847893; doi:10.3389/fnbeh.2020.606900)
Supplement: Supplementary file 5 [file Data_Sheet_4.PDF]

Step 1. Audio Amplifier

For more information see
https://learn.adafruit.com/adafruit-20w-stereo-audio-amplifier-class-d-max9744/overview

Note that our assembly instructions differ from those presented on the Adafruit webpage, and that not all parts in the kit will be used.

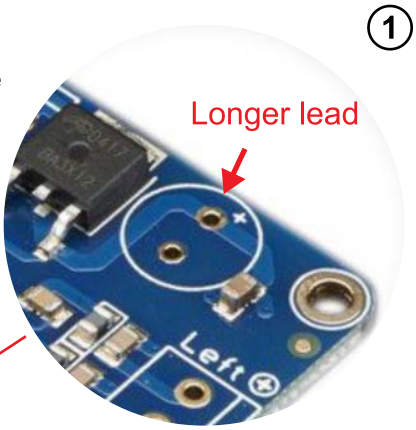

① Solder on capacitor

i) Insert capacitor with the longer lead in the + pad.

ii) Bend the two leads outwards to hold the capacitor flatly in place

iii) Flip the board over and solder both pads. Trim the excess leads

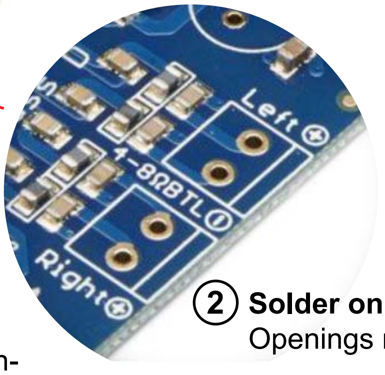

② Solder on transducer screw terminals
Openings must face inward.

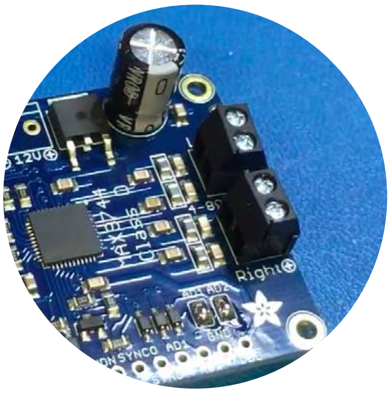

With capacitor and speaker blocks attached

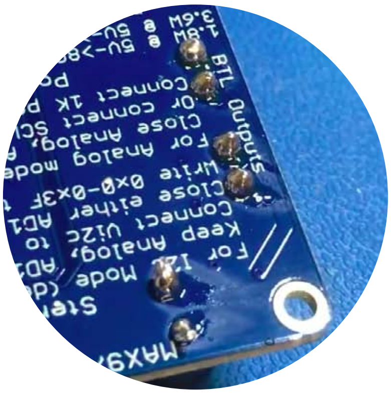

Reverse side showing soldered leads for capacitor and speaker blocks

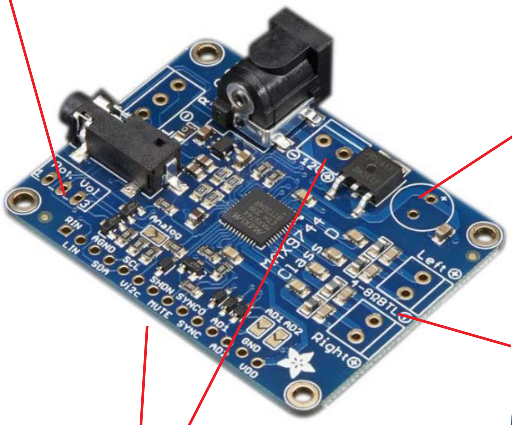

③ Solder on pin headers
Use the custom PCB to ensure proper alignment of all pins:

i) Insert 14-pin header with black plastic on bottom of the audio board, to sit between audio board and PCB.

ii) Insert two single pin headers into the power input pads, to sit between audio board and PCB.

iii) With all pins inserted and aligned, solder pins to the audio board.

④ Switch amplifier to analog mode and attach potentiometer

i) Solder closed the Analog, AD1, and AD2 jumpers.

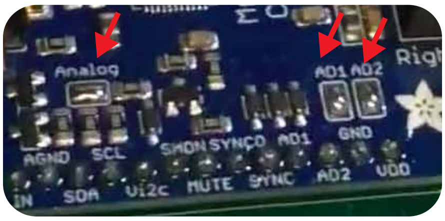

ii) Solder the potentiometer to the three pads labeled “Pot Vol.”

iii) Volume can be controlled by turning the potentiometer knob. Turn the knob to the very right to maximize volume.

Step 2. Teensy

Cut pin header strips to appropriate length for the teensy (24 pins). Using the custom PCB to align the pins correctly, solder the pin headers to the teensy

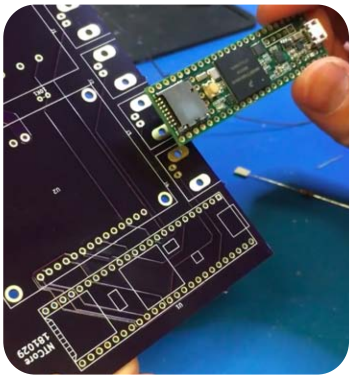
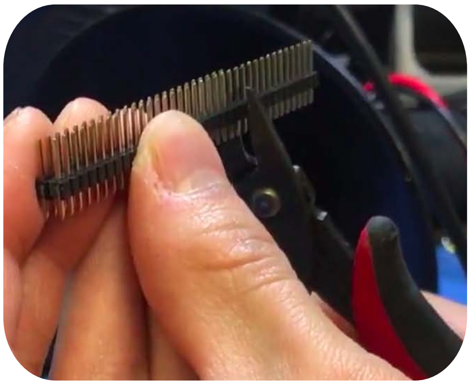
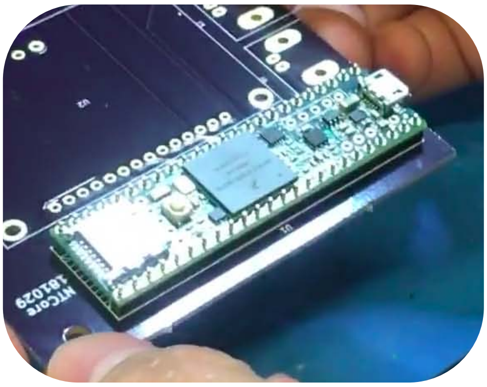

Step 3. Assemble components on PCB

Solder components onto the PCB in this order:
1. Teensy
2. 10k resistor
3. Barrel jack
4. Light panel output screw terminal (with openings facing outward)
5. Audio amplifier
6. BNC connectors
7. Meanwell LDH-45A-500

(1) Teensy: solder all pins to PCB. As with other components, ensure that solder on adjacent pins is not touching.

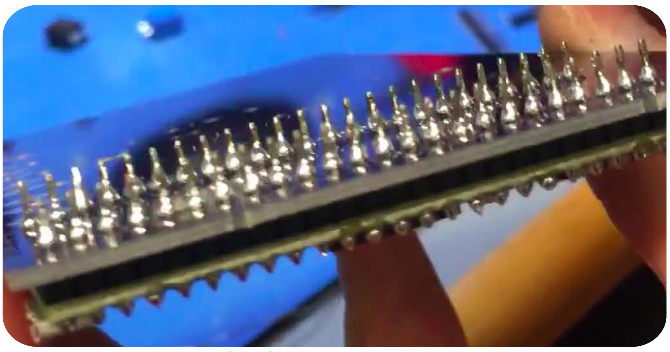

Underside of PCB with soldered teensy

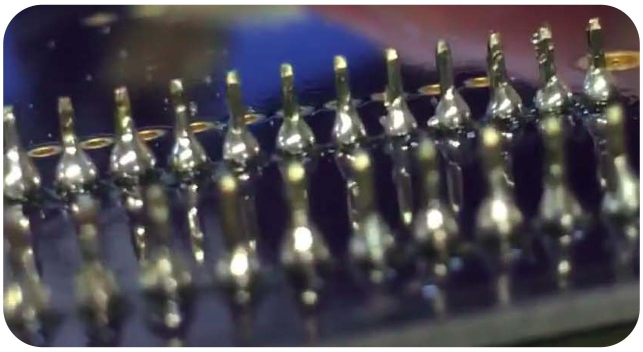

(2) 10k resistor: Bend resistor leads into a U shape as shown below. Insert into the PCB pads labeled “10k,” and bend excess lead outwards to hold in place. Solder to PCB.

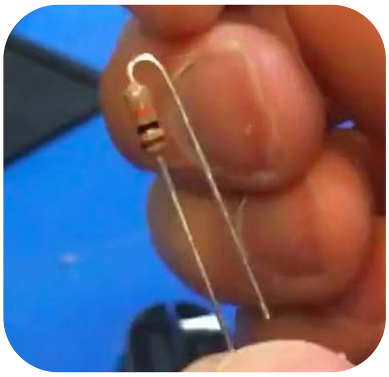
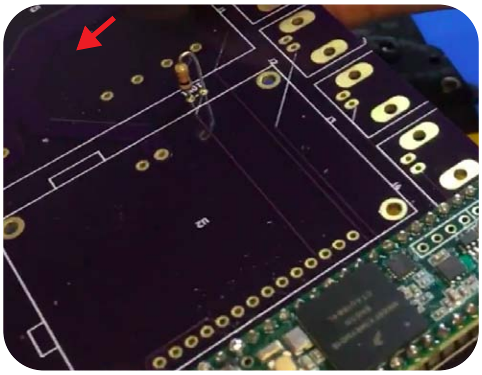
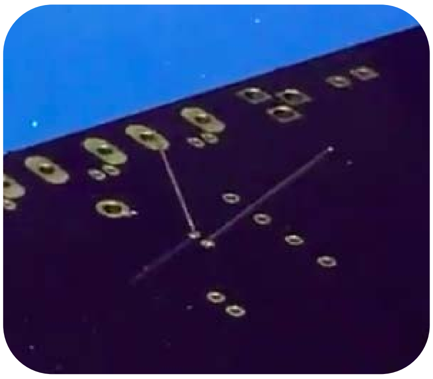

③–⑦ Solder the remaining components onto the PCB

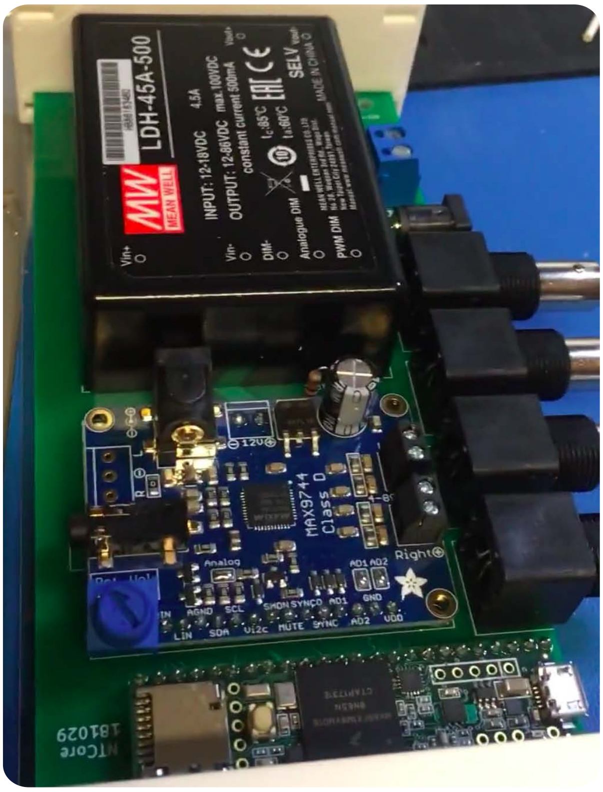

PCB with all components soldered

Step 4. Transducer and light panel wiring

① Transducer wires must be extended to desired length depending on position of transducer and PCB. First, cut desired length of extension wire and feed on tubes of heat shrink.

② Strip the ends of the transducer and extension wires, coil them together, and join with solder.

③ Slide heat shrink over the join, and seal by applying heat from the solder. Avoid burning the coating with excessive heat.

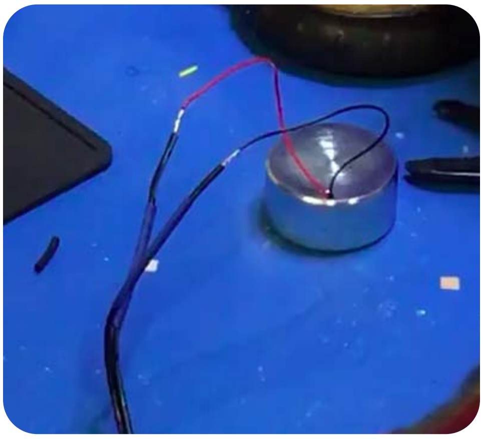

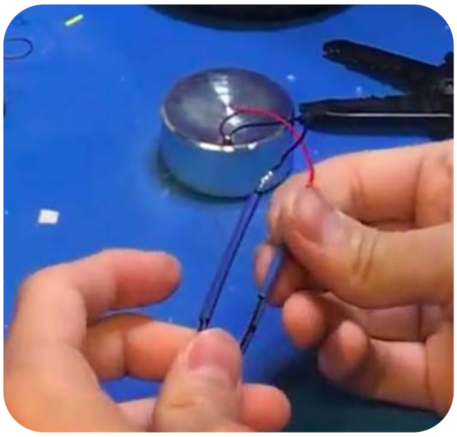

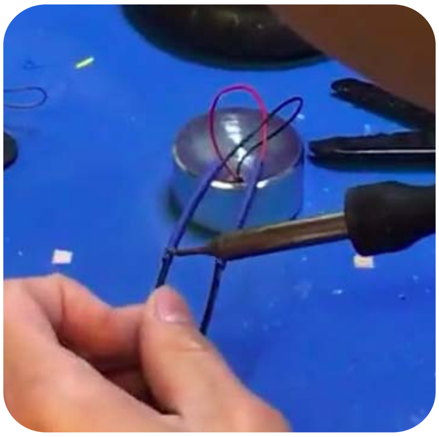

4. Strip other ends of extension wire, and twist each exposed end into a tight braid to avoid fraying/loose ends. Insert each exposed end into a ferrule cap. End of wire should be just visible at the opening of the ferrule tip.

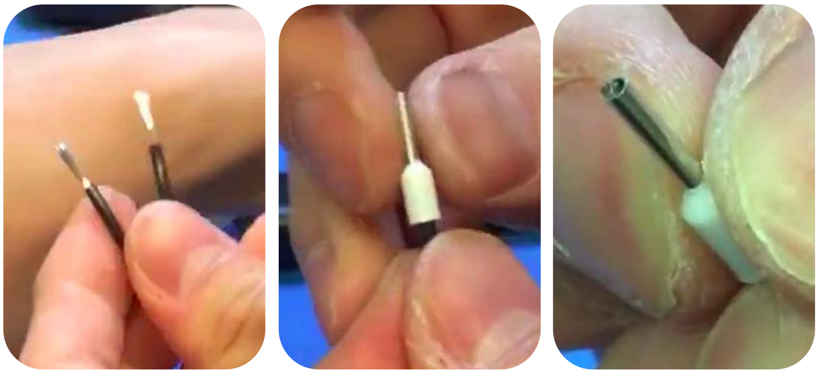

5. Crimp the ferrule caps

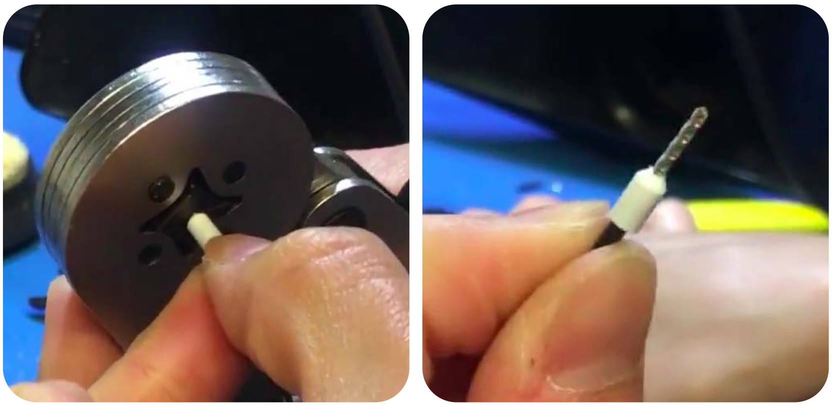

6. Extend light panel wiring as necessary and crimp on ferrules using similar steps as transducer wiring.

!!! Note 1: Transducer and light panel wiring must be fed through the behavior setup shelves/enclosure before being attached to the PCB at the corresponding screw terminals (by inserting into aperture and tightening screws).

!!! Note 2: Ensure proper wire polarity for light panel. The white dashed wire must be attached to the negative (black) wire of the light panel.

For videos depicting the steps above, see supplementary info at https://www.biorxiv.org/content/10.1101/2020.09.08.288621v1
